# An Evaluation of Racial and Ethnic Representation in Research Conducted with Young Adults Diagnosed with Cancer: Challenges and Considerations for Building More Equitable and Inclusive Research Practices

**DOI:** 10.3390/curroncol31040166

**Published:** 2024-04-15

**Authors:** Sharon H. J. Hou, Anika Petrella, Joshua Tulk, Amanda Wurz, Catherine M. Sabiston, Jackie Bender, Norma D’Agostino, Karine Chalifour, Geoff Eaton, Sheila N. Garland, Fiona S. M. Schulte

**Affiliations:** 1 Division of Psychosocial Oncology, Department of Oncology, Cumming School of Medicine, University of Calgary, Calgary, AB T2N 1N4, Canada; 2 Department of Pediatrics, Faculty of Medicine, University of British Columbia, Vancouver, BC V6H 3V4, Canada; 3 Cancer Clinical Trials Unit, University College Hospital, London NW1 2BU, UK; 4 Department of Psychology, Faculty of Science, Memorial University, St. John’s, NL A1C 5S7, Canada; 5 Department of Psychology, University of Calgary, Calgary, AB T2N 1N4, Canada; 6 School of Kinesiology, University of the Fraser Valley, Chilliwack, BC V2S 7M7, Canada; 7 BC Children’s Hospital Research Institute, Vancouver, BC V5Z 4H4, Canada; 8 Faculty of Kinesiology, University of Calgary, Calgary, AB T2N 1N4, Canada; 9 Department of Exercise Sciences, Faculty of Kinesiology & Physical Education, University of Toronto, Toronto, ON M5S 2C9, Canada; 10 Department of Supportive Care, Princess Margaret Cancer Centre, Toronto, ON M5G 2M9, Canadanorma.d’; 11 Young Adult Cancer Canada, St. John’s, NL A1A 5B5, Canada; 12 Discipline of Oncology, Faculty of Medicine, Memorial University, St. John’s, NL A1B 3V6, Canada

**Keywords:** young adults with cancer, psychosocial oncology, race and ethnicity, health equity

## Abstract

The psychosocial outcomes of adolescents and young adults (AYAs) diagnosed with cancer are poorer compared to their peers without cancer. However, AYAs with cancer from diverse racial and ethnic groups have been under-represented in research, which contributes to an incomplete understanding of the psychosocial outcomes of all AYAs with cancer. This paper evaluated the racial and ethnic representation in research on AYAs diagnosed with cancer using observational, cross-sectional data from the large Young Adults with Cancer in Their Prime (YACPRIME) study. The purpose was to better understand the psychosocial outcomes for those from diverse racial and ethnic groups. A total of 622 participants with a mean age of 34.15 years completed an online survey, including measures of post-traumatic growth, quality of life, psychological distress, and social support. Of this sample, 2% (*n* = 13) of the participants self-identified as Indigenous, 3% (*n* = 21) as Asian, 3% (*n* = 20) as “other,” 4% (*n* = 25) as multi-racial, and 87% (*n* = 543) as White. A one-way ANOVA indicated a statistically significant difference between racial and ethnic groups in relation to *spiritual change*, a subscale of post-traumatic growth, *F*(4,548) = 6.02, *p* < 0.001. Post hoc analyses showed that those under the “other” category endorsed greater levels of spiritual change than those who identified as multi-racial (*p* < 0.001, 95% CI = [2.49,7.09]) and those who identified as White (*p* < 0.001, 95% CI = [1.60,5.04]). Similarly, participants that identified as Indigenous endorsed greater levels of spiritual change than those that identified as White (*p* = 0.03, 95% CI = [1.16,4.08]) and those that identified as multi-racial (*p* = 0.005, 95% CI = [1.10,6.07]). We provided an extensive discussion on the challenges and limitations of interpreting these findings, given the unequal and small sample sizes across groups. We concluded by outlining key recommendations for researchers to move towards greater equity, inclusivity, and culturally responsiveness in future work.

## 1. Introduction

Each year, nearly 8000 adolescents and young adults (AYAs; 15 to 39 years of age) are diagnosed with cancer in Canada [1]. With advances in medical treatment, there are a growing number of AYAs that survive and live beyond cancer [1]. While this is encouraging, it is well documented that these AYAs experience significant psychological and social challenges [2], including poorer mental health than the general population. However, AYAs who are from equity-deserving groups are significantly under-represented in the literature [3]. Equity-deserving groups refer to those that experience barriers to equal access, opportunities, and resources due to disadvantage and discrimination [4] and include people that identify as racialized (i.e., persons who are non-Caucasian in race or non-White in colour or Indigenous [5]. A lack of racial and ethnic diversity among AYAs participating in research can have serious research and clinical care consequences. This includes limitations in our ability to generalize study findings, hindering racialized individuals from benefiting from research advances and receiving high-quality care. Building a greater understanding of the psychosocial outcomes of AYAs with cancer from diverse cultural contexts is necessary to be able to alleviate the systemic barriers and challenges faced by this group and advance health equity. An evaluation of the current racial and ethnic representation in AYA cancer research is needed to determine the strengths and gaps in existing research practices.

In this paper, we view race and ethnicity as distinct social constructs that are dynamic and shaped by geographic, cultural, social, and political factors [6]. Race is defined as “a group of people connected by common descent or origin” [7] and ethnicity as “membership of a group regarded as ultimately of common descent, or having a common national or cultural tradition” [7]. In light of the evolving nature of these two terms, and consistent with updated guidance on reporting of race and ethnicity [8], we refer to “race and ethnicity” herein as an aggregate, collective term, while recognizing that there are distinct subcategories of race and ethnicity.

Research on race and ethnicity in relation to the psychosocial outcomes of AYAs diagnosed with cancer is limited. Existing work has focused on children diagnosed with cancer and their families; for instance, a review of the pediatric oncology literature described cultural influences on the healthcare experiences and outcomes of children and families diagnosed with cancer, including those from Asian, White, and Hispanic backgrounds, as well as those placed under “other” backgrounds [9]. Another study examined the health-related quality of life of AYAs on active treatment and found that those identifying as Hispanic experienced poorer physical health than those who were not Hispanic. This sample included AYAs who identify as Hispanic, Black, White, or placed under the “other” category [10]. Although these studies explored ethnic differences in the health and psychosocial outcomes of AYAs, these were secondary analyses to the primary study aims and did not consider comparisons across all ethnic groups. Notably, one study with an ethnically diverse sample of survivors of childhood cancer explored the experience of post-traumatic growth among those from different ethnic groups. The authors found that the experience of post-traumatic growth was lower among those who were Hispanic and primarily spoke English [11]. Furthermore, a systematic review of the mental health of long-term survivors of childhood cancer and young adult cancer identified one study with a large and representative sample showing ethnic differences in the mental health outcomes of AYAs, such that survivors that identified as Black experienced poorer mental health than those that identified as White or Hispanic [12,13]. Finally, there is some research with racial and ethnic minority survivors of young adult cancer that shows that, in spite of poor health outcomes, these individuals experience growth and positive change from their cancer experience [14]. These findings underscore that a complete assessment of one’s cultural background, including one’s racial and ethnic identity, is needed to appropriately tailor care for all AYAs with cancer. However, based on our literature review, studies that focus on evaluating the racial and ethnic representation of AYAs diagnosed with cancer and how race and ethnicity relate to their psychosocial outcomes are evidently sparce and incomplete. Moreover, research that accounts for the psychosocial outcomes of Indigenous Peoples in the AYA literature is near absent.

Despite how little we know about the role of race and ethnicity in the psychosocial outcomes of this group, it is well documented in both Canada and the United States that health disparities exist among racially and ethnically diverse AYAs across the cancer care continuum, from screening and early detection to survivorship [15,16]. It has been suggested that a failure to report on race and ethnicity in health and medical research is problematic and can contribute to challenges related to inequities (e.g., [17]). Furthermore, the exclusion of race and ethnic data from research may mask health disparities [8].

Exploring the associations between race and ethnicity and the psychosocial outcomes of young adults diagnosed with cancer is necessary to elucidate the current challenges and experiences of exclusion encountered by some, as well as the strengths and resiliencies adopted by others. Furthermore, addressing this major gap in the literature can help determine future research aimed at reducing the health disparities experienced by AYAs with cancer and especially those from equity-deserving groups.

## 2. Current Research

We evaluated and described the racial and ethnic representation of a sample from a national, cross-sectional study on Canadian young adults diagnosed with cancer in relation to their psychosocial outcomes.

### Objectives

This study had the following objectives:

(1) Describe the racial and ethnic representation of young adults diagnosed with cancer who participated in a large, national study.
(2) Explore the racial and ethnic differences in the psychosocial outcomes of young adults diagnosed with cancer as an AYA, including post-traumatic growth, psychological distress, social support, and quality of life. Based on the literature we reviewed, e.g., [11,14], we hypothesized that being from a non-White, racial or ethnic group would be associated with greater post-traumatic growth. The other psychosocial outcomes had not previously been studied in this context and were thus exploratory in nature.

## 3. Methods

The current study used observational, cross-sectional data from the Young Adults with Cancer in Their Prime (YACPRIME) study on the longer-term outcomes of young adults affected by cancer, including their psychosocial, physical, financial, and emotional outcomes, compared to their peers without cancer [2,18,19,20]. The YACPRIME study is a collaborative, patient-oriented research project conducted in collaboration with Young Adult Cancer Canada (YACC; [19]), a national organization devoted to young adults >18 years old living with, through, and beyond cancer. The YACPRIME study received ethical approval from the Memorial University Interdisciplinary Committee on Ethics in Human Research (permit # 20180368).

### 3.1. Participants

Participants were eligible to participate in the study if they (1) received a cancer diagnosis between 15 and 39 years; (2) were currently 19 years or older; and (3) resided in Canada. A total of 622 young adults diagnosed with cancer as an AYA completed the YACPRIME study. All participants provided information regarding their race and ethnicity and were therefore included in the final sample for analysis.

### 3.2. Procedure

Participants were recruited from across Canada through advertising, social media, healthcare provider referral, patient partners, and the YACC network. The study was carried out between June 2017 and March 2018. The survey was offered in both English and French. Participants completed an online survey with questions targeting the main objectives of the YACPRIME study.

### 3.3. Measures

**Race and ethnicity.** Race and ethnicity were assessed using a sociodemographic questionnaire developed by the study team. Participants were asked to self-identify their race and ethnicity based on the prompt: “How would you best describe your race/ethnicity?” and with a list of options provided, including: “Aboriginal/Indigenous”, “Arab”, “Black”, “Caribbean”, “Chinese”, “Filipino”, “Japanese”, “Korean”, “Latin American”, “Multi-racial/ethnic”, “Other”, “South American”, “South Asian”, “Southeast Asian”, “West Asian”, and “White”. Participants were able select all that applied to them and/or to self-describe their race and ethnicity using the “Other” option and elaborate using an open-text feature if they desired.

Given the small sample sizes of some of the racial and ethnic groups, responses were organized into four categories for data analysis based on feasibility and parsimony to ensure a more equal distribution of the sample size among groups, including Indigenous, Asian, Multi-racial/ethnic, Other, and White. We acknowledge the challenges and limitations of using collective terms such as “other” in research [8] and discuss the implications of this approach under the “Challenges and Limitations of the Current Work” section.

**Psychosocial well-being**. Psychosocial well-being was assessed using measures of psychological distress, quality of life, and post-traumatic growth.

*Psychological distress*. Psychological distress was assessed using the Kessler Psychological Distress Scale (K10; [21]). The K10 comprises 10 items assessing symptoms of worry, anxiety, and depression experienced in the past month. Participants were asked to rate the extent to which they agree with the statements (e.g., “about how often did you feel depressed?) using a 5-point Likert scale (1 = none of the time; 5 = all of the time). Items were summed to generate a composite score ranging from 10 to 50, with high scores indicating greater distress. Scores ranging from 20 to 24 indicated *mild* distress, 25–29 *moderate* distress, and ≥30 *severe* distress [21].

*Quality of life.* Quality of life was assessed using the 12-item Short-Form Health Survey (SF-12; [22]). The SF-12 is a brief self-report measure of health-related quality of life and comprises 2 subscales: mental (e.g., “have you felt calm and peaceful?”) and physical well-being (e.g., “have you engaged in moderate activities such as moving a table?”). Items were weighted and summed to generate composite scores ranging from 0 to 100. Normative data in healthy adults show a mean of 50 and a standard deviation of 10. Scores > 50 indicated *good* health, 40–49 *average* health, 30–39 *poor* health, and <30 *very poor* health.

*Post-traumatic growth.* Post-traumatic growth was assessed using the 21-item Post-Traumatic Growth Inventory (PTGI; [23,24]). The PTGI is a self-report measure of perceived positive change following a potentially traumatic experience and is composed of 5 subscales: relating to others, new possibilities, personal strength, spiritual change, and appreciation of life. Participants were asked to indicate the degree to which change had occurred in their lives (e.g., “I changed my priorities about what is important in life”) using a 6-point Likert scale (0 = I did not experience this change; 5 = I experienced this change to a very great degree). Items were summed to generate scores for each subscale and a total score, with higher scores indicating greater positive change. The possible score range was from 0 to 105.

**Social support**. Perceived social support was assessed using the 19-item Medical Outcomes Study Social Support Survey (MOS-SSS; [25]). The MOS-SSS comprises 4 subscales, including emotional/informational support (e.g., “having someone to confide in and provide advice”), tangible support (e.g., “having someone to take care of you while you are sick”), affectionate support (e.g., “having someone to love you and make you feel wanted”), and positive social interaction (e.g., “having someone to have a good time with”). Items were summed to generate scores for each subscale and a total score. Raw scores were transformed to scaled scores ranging from 0 to 100, with higher scores indicating better social support. Good social support is defined as scores ≥80.

**Personal and clinical characteristics.** Participants completed demographic questions regarding their age, sex, gender, location (rural, urban, or remote), and total household income as an index of socioeconomic status. They also completed questions regarding their cancer history, including their diagnosis, type of treatment, and time since diagnosis (in years).

## 4. Analysis Plan

All analyses were conducted using SPSS 28.0. We conducted descriptive statistical analyses to describe the racial and ethnic representation of the study sample. Preliminary screening and cleaning of all data were conducted to address any missing data, outliers, multicollinearity, and normality. We performed a one-way analysis of variance (ANOVA) to assess the racial and ethnic differences in post-traumatic growth, psychological distress, quality of life, and social support. We conducted non-parametric, independent-samples Kruskal–Wallis tests to address any violations of homogeneity of variance within the data.

It is important to acknowledge that the sample sizes collected for this study were unequal across the racial and ethnic groups, and particularly small for certain groups. As such, there is likely lower power for any subgroup analyses (i.e., comparisons made with Indigenous, Asians, multi-racial, and “other”). We therefore recommend that readers interpret the results of the post hoc comparisons between these smaller sized racial and ethnic groups with caution and with consideration of the exploratory nature and purpose of this research.

## 5. Results

### 5.1. Participant and Clinical Characteristics

A total of 622 participants were included in the final analysis. The participants included 85% identifying as female (*n* = 537) and 14% as male (*n* = 84), with a mean age of 34.15 years. The participants were also asked to describe their gender identity, with 85% identifying as female (*n* = 530), 13% as male (*n* = 83), and 1% (*n* = 9) “other” gender (i.e., prefer not to answer, transgender, gender queer, or gender fluid). The participants resided across ten provinces and two territories, with participants most commonly from Ontario (*n* = 195), Alberta (*n* = 101), and British Columbia (*n* = 90). A total of 73% (*n* = 456) of the participants reported living in an urban region, 25% (*n* = 157) reported living in a rural region, and 1% (*n* = 9) reported living in a remote region. Moreover, 30% (*n* = 86) of participants reported an average household income of $100,000 or more. Most commonly, 17% (*n* = 106) of participants reported completing treatment within the last 5 years.

Of this sample, 2% (*n* = 13) as Indigenous, 3% (*n* = 21) as Asian, 4% (*n* = 25) as multi-racial/ethnic, and 87% (*n* = 543) of the participants self-identified as White. A subset of participants were placed under the “other” category (3% *n* = 20). Three respondents self-described their racial/ethnic identity as “Jewish,” “Hutterite,” and “Middle Eastern.” A summary of the participants’ sociocultural demographic and clinical characteristics, including disaggregated data for all race and ethnic categories endorsed by the participants, can be found in Table 1.

### 5.2. Racial and Ethnic Differences in Post-Traumatic Growth, Psychological Distress, Quality of Life, and Social Support

For statistical completeness, descriptive statistics (mean, standard deviation, confidence intervals) for key variables, including post-traumatic growth, psychological distress, quality of life, and social support, across racial and ethnic groups (Indigenous, Asian, Multi-racial/ethnic, Other, and White) are reported in Table 2.

The results indicated a statistically significant difference between racial and ethnic groups (Five: Indigenous, Asian, Multi-racial/ethnic, Other, and White) in relation to *spiritual change*, a subscale of post-traumatic growth, *F*(4,548) = 6.02, *p* < 0.001. The post hoc Fisher’s LSD test for multiple comparisons found that participants who identified as “other” endorsed greater levels of spiritual change than those who identified as multi-racial (*p* < 0.001, 95% CI = [2.49,7.09]) and those who identified as White (*p* < 0.001, 95% CI = [1.60,5.04]). Similarly, participants that identified as Indigenous endorsed greater levels of spiritual change than those that identified as White (*p* = 0.03, 95% CI = [1.16,4.08]) and those that identified as multi-racial (*p* = 0.005, 95% CI = [1.10,6.07]). The confidence intervals of these results do not overlap with zero. No statistically significant difference between racial and ethnic groups in relation to the other subscales of post-traumatic growth were observed.

No statistically significant difference was observed between racial and ethnic groups in relation to social support, psychological distress, and quality of life. All the results reported here are summarized in Table 3.

## 6. Discussion

This study examined the racial and ethnic representation of Canadian young adults diagnosed with cancer and how race and ethnicity may relate to psychosocial outcomes, which included an assessment of psychological distress, quality of life, post-traumatic growth, and social support. On the whole, the results highlight an inadequate representation of young adults diagnosed with cancer from ethnoculturally diverse backgrounds, which makes it difficult for us to accurately interpret the findings from the current study. Nonetheless, we observed racial and ethnic differences in some psychosocial outcomes and the research implications for these differences are important to consider for future research and clinical practice.

The majority (87%) of participants identified as White, with ethnic minorities making up 13% of the sample. These proportions are lower than those reported in the national census, which indicates that 27% of the Canadian population are visible minorities [26]. Furthermore, the proportion of White participants compared to other racial and ethnic groups in this sample is consistent with proportions reported in other large-scale, international studies published on children and AYAs diagnosed with cancer, including 86 to 88% White participants in the BRIGHT LIGHT study in the United Kingdom [27,28] and 90% White participants in the Childhood Cancer Survivors Study in the United States [29]. These trends indicate an alarming disparity in the racial and ethnic representation of young adults diagnosed with cancer in research across national and international samples, likely reflecting systematic barriers that exist for young adults diagnosed with cancer from equity-deserving groups to participate in research [30]. Indeed, prior research shows that there is a history of mistrust of the healthcare system experienced by African American adults in the United States and that this mistrust is the primary barrier to research participation [31]. Likewise, Indigenous Peoples have identified the lack of transparency and perceived benefit to their community as challenges in the research experience in Canada [32]. Responding to these limitations by building more inclusive, equitable, and culturally responsive recruitment practices may help to increase engagement from members of equity-deserving groups in research. Prioritizing a research approach whereby people with lived experience are actively involved in the research process [33] may be an integral step towards achieving these goals.

Racial and ethnic differences were observed in some psychosocial outcomes for young adults diagnosed with cancer, but not all. The findings suggest that young adults from equity-deserving groups, including young adults categorized under “other” and identifying as Indigenous, experienced greater spiritual change as part of their post-traumatic growth when compared to those who are multi-racial or White. Past work indicates a similar pattern of results, whereby non-White Hispanic AYAs diagnosed with cancer that spoke Spanish at home reported greater post-traumatic growth than those that spoke English home and non-Hispanics [11]. Our findings suggest that there may be positive change occurring after cancer that may be specific to the experience of equity-deserving groups. Previous studies have found that, for young adults who are newly diagnosed with cancer, spirituality and religiosity are identities that do not fit with the illness experience of these young adults [34]. However, young adults’ self-identities evolve over the course of the illness experience. It is therefore possible that the current results reflect the changing course of spirituality in the cancer journey of young adults. From this perspective, the spiritual change observed in young adults in our sample indicates a positive, protective factor in their psychosocial health. Importantly, past research has not considered the ways in which people from different cultures experience spirituality and spiritual change, and especially those from equity-deserving groups.

Our findings showed that Indigenous young adults with cancer experienced a higher level of post-traumatic growth than those who are multi-racial or White. These results contribute to the growing body of literature on the role of spirituality in Indigenous healing and Indigenous health more generally [35,36]. Of note, a qualitative study with First Nations cancer survivors highlighted that the cancer experience is an opportunity for emotional and spiritual growth that, in turn, enable healing [35]. Our results support this notion that spirituality and uniquely offer evidence of the psychosocial outcomes of Indigenous Peoples who are young adults diagnosed with cancer.

Altogether, our findings offer an important contribution by highlighting differences reported among different sociocultural groups in their experience of spiritual change as part of their post-traumatic growth. This knowledge can inform the ways healthcare providers offer resources, education, and intervention to AYAs from diverse cultural backgrounds in coping with their illness over the course of their cancer journey. It is noteworthy that the implications of this research with Indigenous communities require an understanding and approach grounded in cultural humility and cultural safety, as well as a meaningful and collaborative engagement with Indigenous communities. A discussion of these considerations is outside of the scope of this paper, but we strongly encourage researchers to engage in the necessary learning, reflection, and consultation to conduct community-engaged research with Indigenous communities. Future research explicating the cultural meaning of spiritual change in post-traumatic growth for distinct cultural and Indigenous groups is necessary to better understand what may be culturally specific mechanisms that are contributing to positive change and what may be common to the experiences of all AYAs.

Our study contributes to the literature coming out of the YACPRIME project. While previous efforts by our team focused on the financial, psychosocial, emotional, and physical outcomes of young adults diagnosed with cancer, the current study was unique in focusing on evaluating and describing the racial and ethnic representation of the participants in the project, as well as providing an extensive discussion and reflection on considerations of equity, diversity, and inclusion (EDI). There has been an increasing recognition of the importance of EDI in research and practice. Although our data were collected between 2017 and 2018, the implications of this study still hold relevance, given the lack of historical attention paid to the under-representation of people from equity-deserving groups in research and the importance of analyzing disaggregated data to better understand gaps in our knowledge. Our study was therefore timely to increase awareness of the challenges and limitations of current sampling approaches and to encourage more equitable, inclusive, and culturally responsive research practices for the future.

### 6.1. Challenges and Limitations to Evaluating the Racial and Ethnic Representation of Young Adults with Cancer

We provide here a detailed reflection on and discussion of the challenges and limitations that we faced in evaluating the racial and ethnic representation of young adults diagnosed with cancer in the YACPRIME study.

1. **Unequal sample sizes hinder interpretation of findings**. The current study was composed of unequal samples of young adults diagnosed with cancer coming from different racial and ethnic groups. This meant that interpretation of the racial and ethnic differences found needed to be made with an abundance of caution. There is likely lower statistical power in any comparisons made between minoritized racial and ethnic groups, meaning that the results are likely to be influenced by measurement (random and systematic) error. It is worth noting that, given the exploratory nature of the study, we did not conduct an a priori power analysis to determine whether the results yielded adequate power. Furthermore, descriptive data on the racial and ethnic representation of the sample showed that the majority of the participants (87%) were White, highlighting a clear disparity in the representation of people from equity-deserving groups.
2. **Racial and ethnic groups are not homogenous.** A major limitation of the current research is that participants were asked to self-identify their “racial/ethnic identity,” which merged the two terms. This is a practice that is no longer recommended, as the collective term “race and ethnicity” recognizes that there are distinct/mutually exclusive subcategories within race and ethnicity [9]. Moreover, we organized the participant responses into four major categories of race and ethnicity in order to garner sufficient sample sizes per group for data analysis. This is a limitation because without reporting the specific race and ethnicity of all the participants, we are missing the opportunity to understand important nuances that may exist among diverse AYAs that self-describe their racial and ethnic identities. In a similar way, due to our limited sample, we were unable to conduct further subgroup analysis of the reports of those from more specific racial and ethnic groups, such as those that identify as multi-racial (i.e., the types of multi-racial identities endorsed), hindering our ability to further interpret the perspectives of this racial and ethnic group.
  Organizing racial and ethnic groups under an “other” category has been considered a non-specific and uninformative approach [8]. Our intention in creating this category was twofold: (1) to create larger-sized samples to conduct comparisons in data analysis, which is a common practice [8]; and (2) to allow participants the option to self-describe their racial and ethnic identities rather than endorse the pre-existing options provided in the survey. However, of the 20 (3%) participants that identified as belonging to the “other” category, only 3 participants elaborated on their race and ethnicity through an open-text feature of the online survey. Both measurement and recruitment challenges likely contributed to our difficulty in capturing this important information.
3. **Small sample sizes limit investigation of intersectionality**. We identified racial and ethnic differences in post-traumatic growth and social support. However, due to the small sample sizes across racial and ethnic groups, we did not conduct additional analyses to examine the intersection of race and ethnicity with other sociodemographic factors. Specifically, we do not know whether these observed racial and ethnic differences persist in the presence of other sociocultural factors related to AYAs diagnosed with cancer, such as their age, sex, gender, and socioeconomic status, or factors related to their clinical history, such as years of treatment and type of diagnosis. Given that multiple identities intersect to influence the functioning and well-being of AYAs, incorporating an intersectional lens [37] is necessary to capture the complex and dynamic effects of the sociocultural environment on the well-being of AYAs with cancer. For instance, our sample was skewed towards those identifying as female gender. An assessment of the intersection between race and ethnicity and gender would offer a deeper understanding of the psychosocial outcomes for young adults living with multiple social identities.

### 6.2. Considerations for Future Research: Towards Greater Equity and Inclusive Practices

The challenges and limitations presented inform important recommendations and considerations to enhance equity and inclusivity in future research. We highlight a few key considerations based on our efforts and recognize that there are emerging efforts highlighting similar action plans for future research initiatives (see [38]).

1. **Define and assess race and ethnicity using a standardized, culturally responsive approach.** The lack of a consistent and explicitly stated definition of race and ethnicity can contribute to issues related to construct proliferation and inconsistencies in measurement [39]. Likewise, the absence of a proper definition can obscure other aspects of the sociocultural contexts that may be relevant to the experiences of AYAs from equity-deserving groups, such as experiences of racism and discrimination. A consistent, comprehensive, and culturally responsive approach to defining and assessing race and ethnicity is needed in order to fully capture the role of race and ethnicity, as well as the intersecting effects of race and ethnicity with other sociocultural factors (e.g., age, sex, gender) on the functioning and psychosocial outcomes of AYAs diagnosed with cancer. The development of such an assessment tool would promote a standardized approach to measuring and understanding the multifaceted and dynamic nature of the sociocultural context that shapes the outcomes of this group. The Cultural Formulation Interview [40] exemplifies one tool that has been developed to account for the cultural context in the clinical assessment and treatment of children and adolescents with a range of medical, psychological, and social/emotional challenges. The CFI is increasingly being used in clinical research to explore how culture shapes perceptions towards illness, patient–provider communication, and help-seeking behaviours [41]. At the minimum, we encourage researchers to report in their work the types of questions asked to solicit sociocultural demographic information of study participants to ensure greater transparency and replicability of studies along this line of inquiry.
2. **Adapt and implement culturally safe, inclusive, and equitable recruitment strategies to encourage members of equity-deserving groups to participate in research.** The engagement of AYAs who are underrepresented in the current literature is needed in order to gain a more complete understanding of the perspectives and experiences of AYAs diagnosed with cancer from all sociocultural backgrounds. This knowledge is essential to determine current challenges and barriers that contribute to experiences of health disparity. To do this, we require equitable input from AYAs with lived experience of cancer and who have historically been excluded from research, in adherence with principles of patient-oriented research [33]. Recent efforts have been made in this regard. For instance, a qualitative study was conducted to explore the barriers and enablers for AYAs who have been historically under-represented in cancer research [42]. Preliminary results revealed that some barriers to engagement in research included a limited sense of community, a lack of information, and stigma. Importantly, some of the enablers to research participation included representation and intersectionality approaches. Continued work in this line of inquiry is necessary to amplify the voices of under-represented AYAs and ensure these individuals are effectively included in research along the cancer care continuum. Studies have found that the meaningful engagement of people with lived experience in the research process can be empowering and help build trust between researchers and community [43], as well as affect the quality of care and/or psychosocial support received (e.g., [44]).
3. **Integrate an intersectional lens to examine the effects of multiple and intersecting identities on the functioning and psychosocial outcomes of AYAs with cancer.** There are myriad individual, family, and systems factors that contribute to the disparity in experiences and outcomes of AYAs from equity-deserving groups. At the individual level, as is the focus of the current research, these factors can include but are not limited to age, sex, gender, sexuality, religion, migration status, and class, in addition to race and ethnicity. The study of the social and cultural factors that shape AYA health therefore needs to incorporate the distinct influences of each of these individual diversity factors and their intersection with family and systems factors on functioning and outcomes. Such work requires an intersectional theoretical framework. Intersectionality theory [37] suggests that systems of inequality including those related to race, ethnicity, ability level, and other forms of discrimination can converge, or intersect, to produce unique social dynamics. Past research on the well-being of racialized groups using an intersectionality theory is limited. Integrating this framework would account for the interplay and impacts of race and ethnicity with other social and cultural factors of individuals with intersecting identities and how they overlap with the challenges associated with living with a cancer during young adulthood [45] This approach can potentially help to better identify and address systema tic barriers and problems that can inform future research and policies.
4. **Strive for cultural humility when conducting research and interpretating findings**. Psychological research is predominantly conducted on Western, educated, industrialized, rich, and democratic populations [46]. Likewise, our study was conducted by a group of Canadian researchers living in Canada. In order to ensure the accurate interpretation and generalization of future work, there is a need to recognize that current approaches to research and practice are largely based on Western societal norms and ideals. Cultural humility refers to a lifelong process of self-reflection that may enable researchers to better understand and address health disparities in research [47]. Originally described as a process to enhance awareness of and clinical practice with culturally diverse groups, cultural humility in research involves self-awareness of personal and cultural biases, as well as awareness and responsiveness to cultural issues of others [47]. This practice impacts our engagement in research, interpretation of findings, as well as dissemination of knowledge to diverse audiences.
5. **Promote diversity in representation and team composition**. The current research was unique in that we represented a group of researchers, clinicians, and community partners prioritizing patient-oriented research to study the experiences and outcomes of AYAs with cancer. Research shows that patient-oriented research can rectify power imbalances, promote mutual benefit among patient/community and academic partners, and facilitate reciprocal knowledge translation [48]. We recognize this as a foundational, necessary step to move towards greater equity, inclusivity, and culturally responsiveness in AYA health research. Importantly, there is a growing body of research highlighting strategies that can help enhance patient-oriented research processes, including building trusting relationships with patient/community partners through on-going and direct contact where possible, offering diverse opportunities for involvement, and valuing patient involvement and contribution through proper financial compensation [38,49].

Within the Canadian healthcare systems, high-quality care is meant to be accessible to all people. Health disparities nonetheless exist for people that face various geographic, socioeconomic, and cultural barriers. Furthermore, Canadian society is becoming increasingly multicultural. For these reasons, there is an obligation for researchers to conduct rigorous studies that accurately reflect the diverse Canadian landscape and ensure accessible, inclusive, and high-quality clinical care to all AYAs with cancer. We offer these considerations as a starting point for researchers to reflect on ways we can move towards greater practice of conducting equitable and inclusive research, as well as cultivate a collaborative partnership among researchers and AYAs with lived experience to engage in this critical work. The collective goal is to be able to offer accessible, personalized care that accounts for the intersecting identities of all AYAs impacted by cancer and, in turn, advance health equity in Canada and globally.

## 7. Conclusions

We assessed the racial and ethnic representation of a large, cross-sectional study of Canadian young adults diagnosed with cancer. The results indicated an inadequate representation of young adults diagnosed with cancer from racial and ethnically diverse backgrounds to determine significant and meaningful changes in outcomes among different racial and ethnic groups. We identified major challenges and limitations to conducting this work, including difficulties interpreting unequal samples of participants from equity-deserving groups. We discussed considerations for researchers to conduct more equitable and inclusive research in the future.

## Figures and Tables

**Table 1:** Participant and clinical characteristics.

Participant Demographic Characteristics	*n*	%
**Sex**		
Male	84	13.5
Female	537	86.3
**Gender**		
Male	83	13.3
Female	530	85.2
Others (i.e., prefer not to answer, transgender, gender queer, gender fluid)	9	1.4
Race and Ethnicity *		
Asian	21	3.0
Chinese	7	1.1
Filipino	3	0.5
Korean	1	0.2
South Asian (e.g., Indian)	9	1.4
Southeast Asian (e.g., Vietnamese, Laotian)	1	0.2
Indigenous/First Nations	13	2
Multi-racial/ethnic	25	4.0
Other	20	3.0
Black	2	0.3
Caribbean	4	0.6
Latin American	2	0.3
Other †	10	1.6
White	543	87.0
**Province of Residence**		
Alberta	101	16.2
British Columbia	90	14.5
Manitoba	37	5.9
New Brunswick	11	1.8
Newfoundland and Labrador	66	10.6
Northwest Territories	1	0.2
Nova Scotia	34	5.5
Ontario	195	31.4
Prince Edward Island	5	0.8
Quebec	65	10.5
Saskatchewan	15	2.4
Yukon	2	0.3
**Geographic Region**		
Rural	157	25.2
Remote	9	1.4
Urban	456	73.3
**Participant Clinical Characteristics**	*n*	*%*
Diagnosis Type *		
Breast	170	27.3
Female Genitourinary	60	9.6
Male Genitourinary	9	1.4
Thyroid	45	7.2
Blood	173	27.8
Head and Neck	46	7.4
Gastrointestinal	59	9.5
Skin	18	2.9
Other Types	34	5.5
Multiple Types	8	1.3
**Recurrence or Second Diagnosis**		
Yes	487	78.2
No	135	21.7

* Participants can select all that apply. The total responses in each category may exceed the total sample of participants. † Three participants elaborated on their response under “Others”, including “Hutterite”, “Jewish”, and “Middle Eastern”.

**Table 2:** Descriptive statistics of post-traumatic growth, psychological distress, quality of life, and social support by racial and ethnic group: Indigenous, Asian, Multi-racial/ethnic, Other, and White.

Post-Traumatic Growth		*n*	Mean	SD	95% CI	
Relating to Others	White	480	19.68	8.43	18.92	20.43
	Asian	19	22.47	8.40	18.42	26.52
	Multi-racial/ethnic	20	19.55	9.55	15.08	24.02
	Indigenous	12	18.58	5.20	15.28	21.88
	Others	17	20.41	8.95	15.81	25.01
New Possibilities	White	479	15.23	7.52	14.55	15.90
	Asian	20	18.85	9.17	11.56	20.14
	Multi-racial/ethnic	20	15.20	7.63	11.63	18.78
	Indigenous	12	18.00	4.47	15.16	20.84
	Others	17	14.47	7.73	13.50	21.45
Personal Strength	White	480	11.76	5.12	11.30	12.22
	Asian	20	11.45	6.00	8.64	14.23
	Multi-racial/ethnic	20	11.40	5.36	8.89	13.91
	Indigenous	12	13.00	3.98	10.47	15.53
	Others	17	11.59	5.84	8.58	14.59
Spiritual Change	White	482	3.27	3.53	2.95	3.58
	Asian	19	4.42	3.75	2.62	6.23
	Multi-racial/ethnic	20	1.80	3.17	0.32	3.28
	Indigenous	13	5.38	4.27	2.8	7.97
	Others	17	6.59	3.73	4.67	8.50
Appreciation of Life	White	480	10.34	3.57	10.15	10.66
	Asian	20	9.60	4.27	7.60	11.60
	Multi-racial/ethnic	20	10.35	4.16	8.40	12.30
	Indigenous	12	11.08	2.39	9.56	12.60
	Others	17	12.12	2.76	10.70	13.54
**Psychological Distress**		***n***	**Mean**	**SD**	**95% CI**	
	White	495	24.51	7.99	23.81	25.22
	Asian	20	24.50	8.80	20.38	28.62
	Multi-racial/ethnic	20	27.40	7.18	24.03	30.76
	Indigenous	13	25.00	7.35	20.56	29.44
	Others	18	25.06	7.55	21.03	28.81
**Social Support**		***n***	**Mean**	**SD**	**95% CI**	
Emotional and Information Support	White	461	3.45	1.00	3.36	3.54
	Asian	19	3.61	1.07	3.15	4.07
	Multi-racial/ethnic	19	3.26	1.27	2.80	3.72
	Indigenous	11	3.08	1.12	2.48	3.68
	Others	16	0.26	1.06	2.54	3.54
Tangible Support	White	461	3.73	1.11	3.63	3.83
	Asian	19	3.79	1.13	3.29	4.29
	Multi-racial/ethnic	19	3.76	1.05	3.26	4.26
	Indigenous	11	3.43	0.83	2.77	4.09
	Others	16	3.44	1.37	2.89	3.98
Affectionate Support	White	461	4.02	1.15	3.91	4.13
	Asian	19	3.67	1.34	3.44	4.49
	Multi-racial/ethnic	19	4.09	1.04	3.56	4.61
	Indigenous	11	3.82	0.97	3.13	4.51
	Others	16	3.02	1.61	2.45	3.59
Positive Social Interaction	White	461	3.85	1.04	3.75	3.95
	Asian	19	4.02	1.15	3.54	4.50
	Multi-racial/ethnic	19	3.54	1.06	3.06	4.02
	Indigenous	11	3.21	1.12	2.58	3.84
	Others	16	3.25	1.46	2.73	3.77
Social Support Total Score	White	461	3.68	0.87	3.60	3.76
	Asian	19	3.79	1.00	3.39	4.19
	Multi-racial/ethnic	19	3.54	1.03	3.14	3.94
	Indigenous	11	3.29	0.90	2.76	3.82
	Others	16	3.12	1.22	2.71	3.59
**Quality of Life**		***n***	**Mean**	**SD**	**95% CI**	
Mental Health	White	418	38.95	11.07	37.89	40.02
	Asian	13	35.53	13.75	27.22	43.84
	Multi-racial/ethnic	20	34.88	8.75	30.78	38.98
	Indigenous	12	34.26	8.76	28.69	39.82
	Others	14	42.89	9.40	37.46	48.32
Physical Health	White	418	43.07	9.36	42.17	43.97
	Asian	13	47.37	8.27	42.37	52.37
	Multi-racial/ethnic	20	42.83	11.43	37.48	48.18
	Indigenous	12	46.72	10.47	40.06	53.37
	Others	14	43.15	9.49	33.67	44.61

**Table 3:** One-way ANOVAs to assess racial and ethnic differences in post-traumatic growth, psychological distress, quality of life, and social support.

	Sum of Squares	Df	F	*P*
**Post-Traumatic Growth**				
Relating to Others	167.90	4	0.59	0.67
New Possibilities	172.57	4	0.76	0.55
Personal Strength	23.46	4	0.22	0.93
Spiritual Change	303.71	4	6.02	<0.001
Appreciation of Life	70.41	4	1.37	0.24
**Psychological Distress**	165.50	4	0.65	0.63
**Social Support**				
Emotional and Informational Support	3.47	3	0.90	0.45
Tangible Support	1.84	3	0.48	0.70
Affectionate Support	11.6	3	2.36	0.08
Positive Social Interaction	6.85	3	1.57	0.07 *
Social Support Total Score	4.00	3	1.21	0.19 *
**Quality of Life**				
Mental Health	691.10	3	2.22	0.10
Physical Health	591.07	3	1.91	0.14

* Levene’s test of the homogeneity of variances was significant (*p* < 0.05). Independent samples Kruskal–Wallis Test was performed in lieu of one-way ANOVAs to address the violation of assumptions.

## Data Availability

The data presented in this study is available on request from the corresponding author.
